# Annotations

**Published:** 1902-09-20

**Authors:** 


### Annotations.

### Official Visits to Private Patients.

It is a good rule which has stood the test of ages and is still held in respect by the great mass of the profession that no doctor may interfere with another man's case. But that young and flourishing, and it must be admitted somewhat intrusive branch of medicine which has assumed the title of "Public Health," is like a bull in a china shop, and plays havoc with the proprieties. The medical officer of health is employed, not by the patient spelled with a little "p," but by the People spelled with a very big "P" indeed. He has to perform certain duties, defined more or less vaguely by Act of Parliament, and he relies upon the sanction of law to cover lapses of professional etiquette which in any other person would at once raise a storm of indignation among his professional brethren. He says, with a certain air of logic, "My employer the People have spent endless money in obtaining notification of certain diseases. Am I then when I am told that there are half-a-dozen cases of chicken-pox in a certain house to be debarred from going to see whether these cases may not perchance be suffering from small-pox?" So he goes, or sends his representative; and then the fat is in the fire! "What are you doing with *my* patient?" says the aggrieved practitioner. "What are you doing in *my* patient's house?" is also the cry when the health officer goes there to trace out an epidemic; just as "what are you doing with *my* patient's child?" is said to the public vaccinator when he vaccinates it as by law ordained. According to rule the official doctors are wrong in all these instances, and we need not be surprised at the Birmingham and General Practitioners' Union strongly condemning the action of the Health Committee in arranging for notified cases of chicken-pox to be visited by the medical officers of the City Hospital.

### Finger Prints and the Law.

Considerable interest attaches to a recent trial in which evidence founded upon the now well recognised method of identification by means of finger prints was for the first time accepted and acted upon by an English criminal court. We have not the slightest doubt as to the correctness of the verdict at which the jury arrived, nor can anyone fail to see how powerful a machinery for the discovery of crime the indexing of the finger prints of criminals may become in the hands of an intelligent police. We think, however, that it is worth while to point out that the case tried last week was in no sense a test of the capacity of the system. If on discovering finger prints on the wet paint of the house where the burglary had been committed the police had referred to their collection of the finger prints of habitual criminals, and had by that means been able to point out the perpetrator of the crime, that would indeed have been a triumph. But that was not what was actually done. According to counsel, what happened was that on the name of the prisoner being suggested as that of a person likely to have been engaged in the burglary, the print of this man's thumb, which was in the possession of the police, was compared with the print found on the paint, when "the moment it was examined" its identity with the thumb mark on the wet paint was found to be complete. In the same way both were afterwards found to tally with another print taken after the prisoner's arrest. All of which is very well; we have the expert's word for it, which we accept. At the same time we have a little knowledge of human nature, of the "personal factor" in the collection of evidence, and of the feeling called *esprit de corps*—the tendency of officials to believe, and believing to support, official views—and we cannot but see that in this particular case the jury were compelled to accept blindly and without a chance of further investigation the evidence of experts, and that the experts in similar cases must always, as in this case, be officials, and mostly officials engaged in the prosecution. If the police will find their man by means of finger prints, that will be good evidence; but we should be sorry to see any large extension of this method of corroborating official views by the uncontradictable evidence of official experts.

### A Nice Place to Live In.

It is lucky for some communities that they are not left entirely at the mercy of their legally-elected representatives, and that there exists such an institution as the Medical Department of the Local Government Board to appeal to when other means of redress fail. In the last report of the medical officer to the Local Government Board, an interesting account is given of the state of affairs which was found to exist at Boston, in Lincolnshire, when an inspector was sent down to investigate its general sanitary condition. In this old Sleepy Hollow, notwithstanding that it has a population of some 16,000 persons, and should therefore aspire to possess some of the civilised attributes of a town, it appears that the sewers have been formed by the simple plan of covering over ditches, and generally have been so constructed as to render the complete discharge of their contents impracticable; that the main sewer has been so contrived that a large proportion of its contents is delivered into the river above the town, and so has to pass through the town again on its course towards the sea; that the arrangements for the collection and removal of refuse are so defective that certain of the ash closets were found to be full, "up to and even above the level of the seat"; that in some of the houses "the only way of removing the contents of the privy vault was through the living rooms of the cottages"; that "an intolerable nuisance is created in almost every outskirt of the town by the accumulation of huge heaps of refuse and garbage, deposited by the sanitary authority and allowed . . . to remain for months, pouring out the most sickening smells. During the summer and autumn many houses are almost uninhabitable, being invaded by myriads of flies, which are bred upon these putrid heaps of filth"; that a farmhouse is the sole accommodation for infectious sickness in this and in neighbouring districts; that there is no disinfecting apparatus; that the by-laws are out of date, while those which exist are not enforced; and that "the Boston Town Council is apparently concerned rather in furnishing plausible excuses for its shortcomings than in setting to work to improve the conditions of its district. Clearly this Boston is a nice place to live in.

